# A Low-Cost Optical Remote Sensing Application for Glacier Deformation Monitoring in an Alpine Environment

**DOI:** 10.3390/s16101750

**Published:** 2016-10-21

**Authors:** Daniele Giordan, Paolo Allasia, Niccolò Dematteis, Federico Dell’Anese, Marco Vagliasindi, Elena Motta

**Affiliations:** 1 GeoHazard Monitoring Group—Institute of Research for the Geo-Hydrological Protection—National Council of Research of Italy, 10135 Turin, Italy; daniele.giordan@irpi.cnr.it (D.G.); paolo.allasia@irpi.cnr.it (P.A.); federico.dellanese@irpi.cnr.it (F.D.A.); 2 Fondazione Montagna Sicura, 11013 Courmayeur, Italy; mvagliasindi@fondms.org (M.V.); emotta@fondms.org (E.M.)

**Keywords:** image matching, glacier kinematics, low-cost equipment, monitoring, remote sensing

## Abstract

In this work, we present the results of a low-cost optical monitoring station designed for monitoring the kinematics of glaciers in an Alpine environment. We developed a complete hardware/software data acquisition and processing chain that automatically acquires, stores and co-registers images. The system was installed in September 2013 to monitor the evolution of the Planpincieux glacier, within the open-air laboratory of the Grandes Jorasses, Mont Blanc massif (NW Italy), and collected data with an hourly frequency. The acquisition equipment consists of a high-resolution DSLR camera operating in the visible band. The data are processed with a Pixel Offset algorithm based on normalized cross-correlation, to estimate the deformation of the observed glacier. We propose a method for the pixel-to-metric conversion and present the results of the projection on the mean slope of the glacier. The method performances are compared with measurements obtained by GB-SAR, and exhibit good agreement. The system provides good support for the analysis of the glacier evolution and allows the creation of daily displacement maps.

## 1. Introduction

During the last century, global warming has caused a great retreat of glaciers, especially in the Northern Hemisphere [1]. Glaciers have an important impact on population because they store fresh water, thereby affecting agriculture, water management and hydroelectric power plants [2]. This trend has increased the frequency of glacier-related instabilities, which constitute a major concern in mountain areas, in particular in the Alps, where they can interact with densely populated areas [3,4,5]. An important example of these processes is the break-off of temperate or polythermal glaciers, resulting from a combination of geometric, thermal and water flow conditions. Unfortunately, these conditions are still poorly understood, making prediction of break-off events difficult. This is partially due to the lack of systematic long-term observations of temperate glaciers and to the difficulty of monitoring this particular geomorphological process. 

The use of monitoring system for the study of glaciers is often focused on the definition of long-term evolution through periodic acquisitions of remotely sensed data [6,7,8]. These applications, usually combined with the use of in situ devices, have been adopted to periodically acquire the geometry of the ice mass and to evaluate its changes [9].

Approaches for the remote sensing monitoring of glaciers include: (i) SAR interferometry [10,11]; (ii) amplitude tracking [12,13]; (iii) LiDAR surveys [14,15,16]; (iv) multispectral image analysis [17]; (v) photogrammetry [18,19]. The major constraints of these methods are the long revisit times of the airborne and satellite applications and the high cost in terms of economic and logistical resources related to long-lasting surveys involving devices such as ground-based synthetic aperture radar (GB-SAR). From this perspective, the application of monitoring systems must be able to offer a high acquisition rate with an approach very similar to the one used in landslide monitoring [20,21,22,23]. 

Due to recent hardware and software improvements in the field of photogrammetry, the use of computer vision applications can be now considered a new effective approach for the monitoring of the evolution of gravitational processes using low-cost equipment [24]. The use of photogrammetric systems was initially tested with satellite images for landslide monitoring [25] and in other contexts, such as glaciers [18,19] or river reaches [26,27]. 

Jiang et al. [28] developed one of the first ground-based applications of close-range photogrammetry, which ensures a much finer resolution than other approaches. To the authors’ knowledge a few studies have been published using this approach for monitoring glaciers [29,30,31].

Ground-based optical systems are often composed of one or more stations acquiring a sequence of high-resolution photos of the studied area, and these photos are then processed using different approaches. One possible method is stereoscopy, which is usually based on the creation of a 3D model based on the simultaneous acquisition of two images of the same target from two acquisition points [30,32]. Other possibilities include the use of a single camera for a multi-temporal analysis based on image matching [29,33], change detection [34], pixel tracking [31,35], and, in recent years, UAV-borne photogrammetry for operative purposes [36,37].

One of the added values of these techniques is the possibility of obtaining a dataset of optical images that can be used not only for numerical analysis but also for morphological analysis of the studied phenomenon [38,39]. A photo sequence of the evolution of a geomorphological process can often be very useful for understanding the dynamics that control and characterize its evolution. 

The aim of the present work is twofold: firstly, the technological development of an operational, low-cost, optically-based monitoring system that is robust enough to resist the extreme weather of the Alpine winter at high altitude; and secondly, the development of software for processing the acquired data to estimate the glacier surface deformation. 

In this paper, we present the design of an experimental application of an optical system based on image cross-correlation [34,40,41], hereafter called the Pixel Offset (PO) method, developed for monitoring the evolution of the Planpincieux glacier, on the Italian side of Mont Blanc (Aosta Valley Region, NW Italy). The research project started in September 2013 and remains active, continuously acquiring photographs. The principal steps presented here are as follows: (i) the development of a methodology for the acquisition and selection of images; (ii) the identification of a methodology for morphological analysis; (iii) the application of the PO method.

The paper is organized as follows: in Section 2, the study area is presented. In Section 3, the installed ground-based equipment and the features of the consumer-grade camera are described in detail, with some discussion of the ancillary radar surveys. In Section 4, we present the operative methodology of the long-term monitoring programme, from the acquisition to the data processing. In Section 5 and Section 6, the results and discussion are presented. Our conclusions follow.

## 2. Study Area

The Aosta Valley Region is located in north-western Italy and is characterized by the presence of the highest mountain in the Alps. The Planpincieux glacier (located at approximately 45.85° N 6.97° E) is in the Ferret Valley (Aosta Valley) on the south side of the Mont-Blanc Massif in the Italian Alps. This glacier is within the open-air laboratory of the Grandes Jorasses and is part of the Grandes Jorasses—Planpincieux glacier composite, lying at elevations between 3700 and 2530 m asl and covering approximately $1\;{\mathrm{km}}^{2}$. 

The glacier is composed of two different ice streams, starting at 3700 and 3300 m asl, and merging into a wide plateau (15° slope) between 2950 and 2900 m asl. Below this altitude, the steep tongue of the glacier starts (32° slope), and reaches an elevation of 2530 m asl.

**Figure 1:**
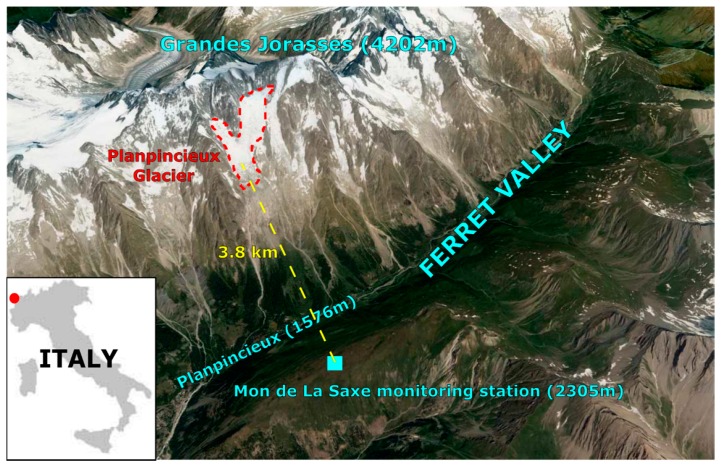
Aerial view of the Ferret Valley. The Planpicieux glacier is located in the upper part of the image. In the bottom part of the Ferret Valley, the Planpincieux hamlet represents the main element at risk of possible ice avalanche activation. On the opposite side of the valley, at a distance of 3.8 km, the monitoring station is located on the top of Mon de La Saxe.

**Figure 2:**
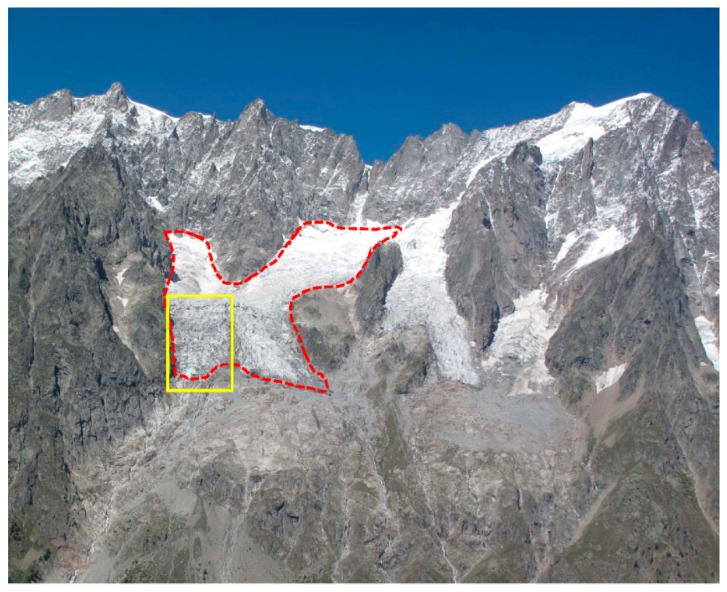
Overview of the Grandes Jorasses massif (northern sector of the Mount Blanc massif) with the Grandes Jorasses Glacier (on the right side of the image) and the Planpincieux Glacier (highlighted in dashed red line). The image was acquired by the webcam of the monitoring station. The yellow box indicates the part acquired by the ZOOM camera, which is the most active region of the Planpincieux glacier.

The glacier tongue is intensely crevassed. Based on the crevasse pattern, the glacier tongue appears to be separated into two different ice flows by a central ridge in the bedrock. The eastern side of the glacier margin lies on an open slope (Figure 1), while the westernmost side (in yellow in Figure 2) is confined by a steep rock wall and lies just above a significant increase in the bedrock slope. This part of the glacier fits the definition of a “*terrace avalanching glacier*” [42]. This part of the glacier is the most active, and the paper focuses on the study of the western tongue. The entire glacier can be classified as a “*temperate steep glacier*” [43].

Historical photos show that this rock-step has always interrupted the glacier flow, even when the terminus of the glacier was hundreds of metres down-valley. Therefore, the westernmost side of the glacier margin can be considered a single unit with its own dynamics. The temporal analysis presented in this paper focuses on this single unit. 

A long series of historical instabilities affecting the glacier is recorded. The recurrent processes consist of floods or hyper-concentrated flows triggered by outbursts of glacial water pockets. Such events were recorded in 1929, 1984, 1985, 1986, 1987, 1996, 1998 and 2008. Floods or flows usually cause damage to the road at the bottom of the valley and to the bridge on the Montitaz stream. In some cases, the flood waves propagated over 10 km downstream.

In October 2011, a large crevasse opening was observed in the westernmost part of the glacier margin. This crevasse, which was considered anomalous, sealed later in the next month and then appeared again for a few months in September 2012. Nevertheless, because the glacier has been categorized as potentially dangerous in the frame of a monitoring plan for glacier-related hazards (set up by the regional authorities and managed by Fondazione Montagna Sicura, FMS), this event raised some concern. Historical data describe some mixed ice-snow avalanches that were triggered by the glacier and reached the valley bottom. The most important of these events occurred in December 1952. 

Therefore, we decided that the Planpincieux glacier could represent an interesting case study in order to better understand temperate glacier dynamics and detect possible instability indicators. 

## 3. Dataset and Equipment

In the following sections, we illustrate the optical monitoring system developed for the evaluation of the glacier deformation using PO analysis. Moreover, we present the radar surveys performed for comparison with the PO results and a LiDAR campaign.

### 3.1. Monitoring Station

The aim of the project was to produce a monitoring system able to capture the evolution of the glacier over time, but the choice of the type of the monitoring system has been conditioned by some critical issues: (i) the steepness of the glacier and surrounding rock walls and the highly crevassed area make difficult to install in situ monitoring systems; (ii) the high evolution rate of the glacier and the frequent icefalls hinder the installation of in situ measurement targets (e.g., topographic benchmarks or GPS); and (iii) the only possible sites for the instrumentation are the valley floor and the opposite side of the valley. Hence, remotely sensed systems have been judged to be the only possible solution for quantitative measurements. 

LiDAR and GB-SAR have been considered too expensive for long-term installations and consequently were only used for short campaigns to support the study. After considering numerous remote sensing systems, we decided to construct a low-cost monitoring station based on the acquisition of optical images. The stereoscopic photogrammetric approach, which requires the installation of at least two different acquiring cameras in different locations, was excluded for logistic and financial reasons. A single-camera system that can be used for the detection of movement orthogonal to the line of sight was considered the most suitable solution.

For the identification of the location of the monitoring station, the main movement direction of the glacier was first estimated in order to acquire the largest motion component as possible. Two possibilities were considered: near Planpincieux village at the bottom of the valley and on top of Mon de La Saxe. The monitoring station was installed at the end of August 2013 on the opposite side of the valley, on top of Mon de La Saxe because this positioning ensures good visibility of the entire glacier (Figure 1). The mean distance between the monitoring station and the glacier is 3.8 km, which is longer than the distances typically associated with other similar ground-based applications [29,30,44]. The choice of Mt. de La Saxe produces several accessibility problems, especially during the winter season when the trail is at risk of snow avalanches. This problem of accessibility has necessitated the use of a remote controlled system to reduce the need for direct access to the station. 

**Figure 3:**
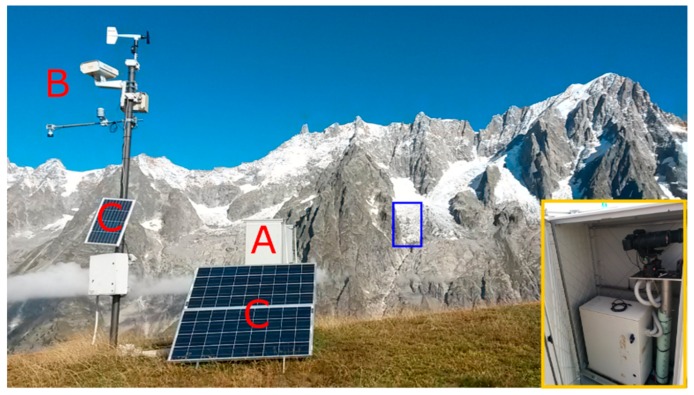
Monitoring station: (A) Box containing the two cameras. (B) Webcam and weather station. (C) Solar panels. The area acquired is highlighted in blue. In the yellow box: detail of the acquisition system within the shelter box.

We developed a monitoring station composed by different modules (Table 1, Figure 3). The first module is hosted by a plastic box and contains a EOS 600D DSLR camera (Canon, Tokyo, Japan; http://www.canon.it/for_home/product_finder/cameras/digital_slr/eos_600d/) (hereafter called ZOOM) with a DG-OS HSM lens (Sigma, Ronkonkoma, NY, USA) and all the relevant hardware. Additionally, a similar camera (WIDE) with a small focal lens (capturing a larger field of view) is also installed in the shelter box. The optical zooms are manually set and mechanically locked. The ZOOM observes the most active part of the glacier, while the WIDE camera acquires images of the entire glacier. The focus is set at infinite, with a hyperfocal distance of approximately 500 m. This set-up ensures the observed part of the glacier is always in focus.

Another module is mounted on top of a 3-m-high pole, which hosts a webcam that acquires medium-quality images of the glacier throughout the year. The use of the pole ensures the possibility of acquiring images during the winter season, when the box can be covered by snow. Figure 2 shows an example of a picture taken by the webcam, and the yellow box indicates the area acquired by ZOOM, which corresponds to the most active part of the Planpincieux glacier. 

We compared the results of the installed cameras with calibrated cameras under laboratory conditions to evaluate the performances of the image correlations. The errors were some orders of magnitude lower than the measured data. Thus, we decided to not calibrate the cameras, as the committed error is much lower than the system precision.

The monitoring station also includes (i) a low-power PC; (ii) a UMTS modem; (iii) a battery pack and (iv) a solar power supply. All these components are stored inside a plastic box mounted to four supports cemented into the ground. To minimize the effect of the vibration caused by the wind (which can be very strong especially during the winter season), the two cameras are mounted to a pole cemented into the ground and separated from the external box.

The ZOOM and WIDE systems are able to acquire a picture every 2–3 min and to transfer the images to the server of the National Council of Research (CNR) in Torino, where they are stored and analysed. The frequency of the acquisition can be remotely controlled and depends on the glacier activity. During the summer, when the movements and number of icefalls are high, the acquisition frequency is set to 30 min. During the other seasons, when the activity of the glacier progressively decreases, the frequency is set to one hour. Since the system acquires RGB images, the acquisition is limited to the diurnal part of the day. 

### 3.2. LiDAR and GB-SAR

As mentioned before, the use of LiDAR and radar has been evaluated but considered not suited for long-term acquisitions. To support the optical system with a high-resolution digital terrain model (DTM) and to have data collected via different technological methods that are able to measure the glacier movements, LiDAR and radar surveys have been performed. 

The airborne LiDAR survey was performed on 9 June 2014, and a high-resolution DTM has been produced. In 2015, during the period between 2 September and 15 October, a radar campaign with an Ibis-S ground-based synthetic aperture radar (GB-SAR) was performed from the Planpincieux hamlet. 

The data have been processed by the author using an interferometric approach [10,11,45]. The time separation between consecutive acquisitions is 16 min. Pixels with a Mean Coherence (MC) lower than a given threshold (MC<0.6) were excluded from the processing. The atmospheric noise (Atmospheric Phase Screen, APS) was computed using fixed pixels, identified by Amplitude Dispersion, DA<0.35. APS was estimated by fitting a 2D linear model [46] and was then subtracted from the interferometric phase. The unwrapping algorithm [47] was applied before and after the APS filtering in order to correct possible errors introduced during the APS subtraction. Finally, the results were georeferenced, and the actual metric displacement was estimated by projecting the measured deformation along the steepest gradient direction [11].

The results of the radar survey in 2015 will be described in detail in a future paper. However, some results are included in this study and compared with the results of the PO analysis.

## 4. Methodology

**Figure 4:**
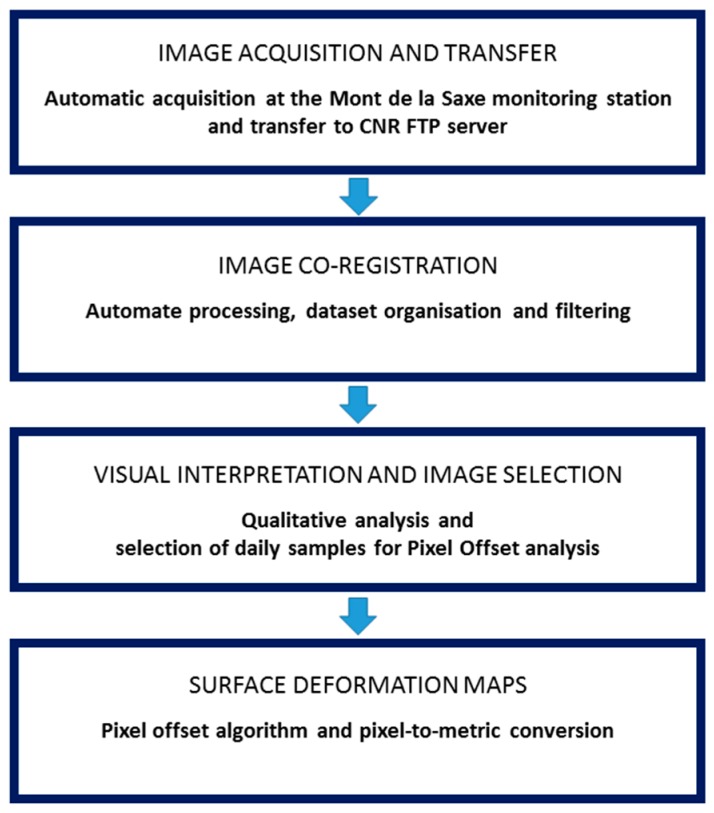
Work flow of the image processing.

This chapter focuses on the monitoring chain, from the data acquisition to the processing and PO application. The workflow identifies four main steps: (i) data acquisition and transfer; (ii) co-registration; (iii) visual analysis and image selection and (iv) Pixel Offset analysis. The scheme of the method is illustrated in Figure 4. This procedure has been developed and tested on the area monitored by the ZOOM camera, which is the most active part of the glacier. Future applications will be extended to the dataset collected by the WIDE camera, which monitors the entire lower part of the glacier.

In the following, we present the different steps of the methodology and the results obtained based on the analysis of the available dataset. Hereafter, all the data are from the ZOOM system.

### 4.1. Image Acquisition and Transfer

The monitoring station has been designed to acquire and transfer the images to CNR servers. The system can be remotely controlled to define the target area and the frequency of acquisition of images to ensure an accurate description of the glacier evolution. 

The images have been continuously captured since August 2013. The acquisition system has been demonstrated to be rugged enough to withstand poor weather conditions and has produced a dataset containing sufficient images for the application of the method, ensuring a nearly continuous monitoring of the glacier movement. 

The monitoring system has acquired and transferred more than 15,000 images from September 2013 to June 2016. The number of days in which the ZOOM system acquired images corresponds to approximately 63% (Table 2) of the considered period. 

The remaining 37% represents cases in which there were exceptional weather events (e.g., very strong wind or abundant snowfall) or problems with the power supply due to the insufficient production of power by the solar panels due to snow cover. Technical problems were related to improvements and maintenance of the system, which caused a system stop. If we consider the entire dataset, 40% of the images were acquired in ideal conditions (absence of clouds in the field of view during daytime). 23% of the images were captured correctly but with poor visibility (issues related to cloud cover, shadows, snow cover, and atmospheric dust).

### 4.2. Co-Registration

The system has been configured to minimize the movement of the camera and to always acquire the same area. However, several factors, such as fluctuations in temperature and humidity and the effect of air refraction, can create some shifts between pictures. The first step for the application of the pixel offset algorithm is image co-registration to obtain perfectly superimposed images. The co-registration algorithm achieves image registration by normalized spatial cross-correlation with sub-pixel precision [40,48].

**Figure 5:**
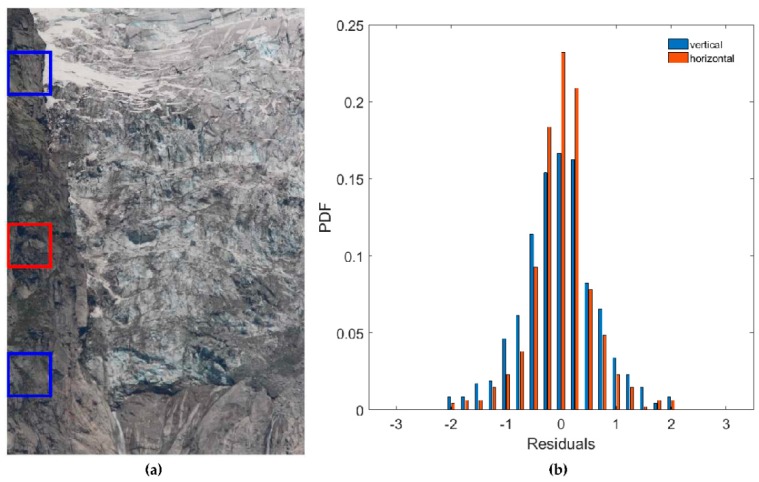
(**a**) In red the reference window used for the co-registration process, lying on the rock outcrop. In blue the two windows used for error estimation; (**b**) Frequency distributions of Mean Absolute Error computed separately for the vertical (blue) and horizontal (orange) dimensions.

The cross-correlation of each pair of images is computed in a reference tile, which is assumed to be time-invariant (i.e., it does not change over time due to movement and/or deformation). In this study, the reference tile uses a portion of the bedrock outcrop characterized by a steep slope in order to limit the incidence of snow cover and shadows and to minimize optical distortions due to the photographing of sloping surfaces (Figure 5a). Cross-correlation is computed to define the 2-dimensional shifts necessary to exactly superimpose the first picture (Master) on the second picture (Slave). The Slave image is then shifted to achieve superimposition. 

The co-registration is performed automatically by a software package that verifies the coherence (see Section 4.4.1) between images. Moreover, a counting of the RGB colours is performed as a further control on image quality. The system discards images that do not satisfy the given conditions.

Environmental conditions, such as particles, air refraction and light conditions, can affect result precision, causing a loss of image sharpness and scaling phenomena. For these reasons, an error evaluation is recommended.

In this study, the residual errors after co-registration have been estimated as follows: two different windows of similar size are defined on the top and at the bottom of the outcrop (Figure 5a). After the first co-registration step, cross-correlation is computed again between the respective windows of the co-registered images. The frequency distributions of the resultant 2-dimensional shifts obtained by the analysis of more than 300 images (i.e., one image per day) are shown in Figure 5b with respect to both the horizontal and vertical dimensions. The Mean Absolute Error (MAE) is computed and is assumed to be the residual error of the co-registration application. The obtained MAE along the horizontal dimension is 0.92 pixels (px), and the MAE along the vertical dimension is 0.76 px. The different amounts of the MAE along the two directions can be due to different pixel metric dimension (i.e., the Ground Sampling Distance, GSD). In fact, the reference tile features a very steep but not vertical rock wall, hence, the vertical GSD is larger than the horizontal (as explained in Section 4.4.2).

Based on the error distributions, in the current work a value of ±1 px has been considered to be the system precision in both dimensions. Therefore, in this study the resulting shifts are rounded at the nearest integer. This choice is supported by two factors: (i) the value of the MAE, that is on the order of 1 px and (ii) based on a posteriori observations, the measured glacier deformations are quite large (on the order of a few pixels/day) and a sub-pixel offset estimate is not necessary.

This process has also demonstrated the non-occurrence of rotational effects due to camera vibrations for the frequency distributions of residuals are approximately symmetric (Figure 5b). Other more complex optical aberrations could be present, but working only in differential mode (i.e., comparing images taken by the same camera), these geometric distortions do not affect the results of the pixel offset algorithm.

### 4.3. Visual Analysis and Image Selection

Image analysis techniques can provide an objective, automatic and quantitative description of changes in selected scenes, thus avoiding uncertainties and inaccuracies due to the sensitivity of the operator, as well as considerable time-consuming work [34]. Nevertheless, a thorough analysis with the human eye still remains essential to the interpretation of changes in glacial dynamics (i.e., major icefalls and all the morphological changes), even with a qualitative approach [38,39].

The visual interpretation procedure has been carried out on the co-registered images for the entire dataset. This analysis involves the following steps:

- Manual selection of one image per day, ideally taken at the same hour (approximately midday) in order to have the same light conditions. When weather conditions make this impossible, the closest available image is chosen.
- Superposition of a reference grid on each image in order to detect, localize and describe the analysed processes.
- Visual comparison between images (one-to-one, each day with the previous) in order to detect sudden changes such as icefalls, sudden changes in water discharge, etc.
- Editing of a sequence of images covering the entire dataset period; this allows to detect long-term and slow processes, such as continuous displacement or crevasses widening.

Basically, this qualitative analysis focuses on four type of processes: (i) glacier flow; (ii) icefalls; (iii) crevasse opening and widening; and (iv) water discharge.

#### 4.3.1. Glacier Flow

**Figure 6:**
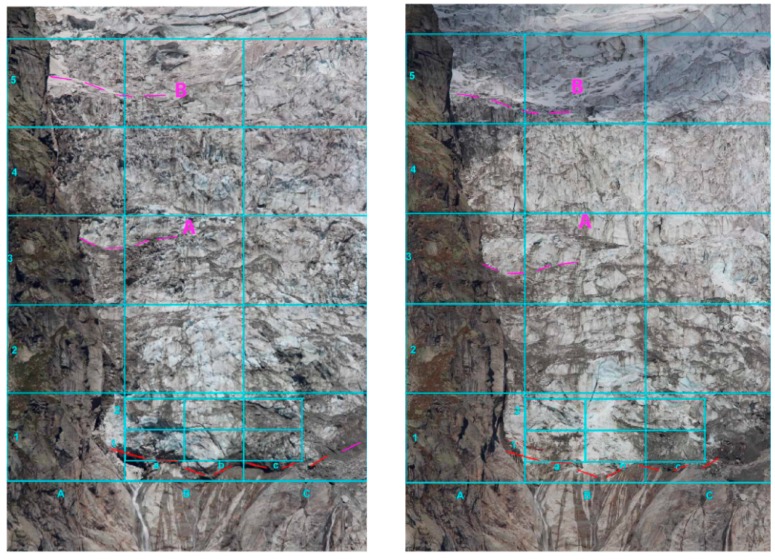
Visual analysis of the glacier flow on 2 August 2015 (**left**) and and 8 October 2015 (**right**). The superimposed grid simplifies the comparison between different areas. In particular the movement of the pattern highlighted by the letter A appears evident. The B pattern appears less changed.

The analysis of the glacier flow has been conducted by focusing on recognizable features of the glacier surface, such as crevasses with a well-defined shape, fine debris spots, or ice surfaces bounded by two crevasses. The superimposition of a regular grid on the images allows the comparison of the flow features between different areas. In Figure 6 the images of 2 August 2015 and 8 October 2015 are shown, and it is evident that considerable displacement occurred in the right part. 

#### 4.3.2. Icefalls

Although the glacier flows downstream with discrete velocity, the front remains almost at the same position throughout the year, just above an area with a steeper slope. When the glacier oversteps the slope break, the flow is balanced by an increase in the frequency of small icefalls from the glacier margin. 

#### 4.3.3. Crevasses Opening and Widening

Qualitatively, the flow movement seems to be balanced both by new fracturing or widening of existing crevasses and by ice deformation. However, the first process is likely the most relevant. The lower part of the glacier has a stair-shaped morphology, i.e., steep ice walls are separated by major fractures, probably following the bedrock shape. The opening and widening of crevasses causes the ice walls to slide down, allowing them to maintain their shape for a long period. In some cases, the glacier has experienced sudden widening of crevasses, causing the lower block to move down quickly, or even to collapse.

#### 4.3.4. Water Discharge

Water circulation and pressure at the glacier/bedrock interface can significantly affect glacier stability. Therefore, changes in water discharge from the glacier have been observed, as they may indicate changes in the subglacial or englacial flow. In the studied images, only great changes in flow rate can be observed, as well as shifts in the stream position. Of course, seasonal variation in flow rate is normal and must be taken into account.

Although water traces are detected below the glacier front, the stream coming from the right part of the glacier mainly remains at the extreme right side, and it flows into a channel in the rock wall below the glacier itself. In just few cases, water discharge has spread along the entire front, e.g., in spring and early summer 2014. No anomalous changes in flow rate were detected during the observed period (i.e., sudden decreases or increase in the flow or sudden changes in the position of water flow).

#### 4.3.5. Glacier Sectors

From the visual analysis, different sectors of the glacier have been broadly identified, based on particular flow, collapse and crevasse behaviours. From the bottom to the top of the image: (i) the *front sector* features an ice wall lying on a very steep bedrock formation partially covered by the vertical front of the glacier and is subjected to frequent ice-falls; (ii) the *crevasse sector* is characterized by the highest velocity and by the formation of crevasses which causes entire ice walls to slide down; (iii) the *middle sector* also exhibits high velocity but the flow is more homogeneous; and (iv) the *plateau sector* is located in the uppermost part and is plateau-shaped.

### 4.4. Surface Deformations Estimation

The Pixel Offset method aims to determine information on shifts in position orthogonal to the line of sight (LOS) by comparing two co-registered images captured at different times [29,35]. The PO method is commonly used in remote sensing to determine superficial displacements on images acquired at different times [41]. In this study, the use of this application for the measurement of the glacier surface movements is possible due to the availability of a dataset composed of high-resolution images. The PO method is applied to pairs of co-registered images called Master and Slave. A two-dimensional displacement map is the result of the cross-correlation process between each Master/Slave sample. To produce useful information for interpretation, the discrete results are visualized as LOS-orthogonal displacement maps overlaid to the Master image, with the convention that the y-axis is positive downward and the x-axis is positive towards the right side of the image. 

**Figure 7:**
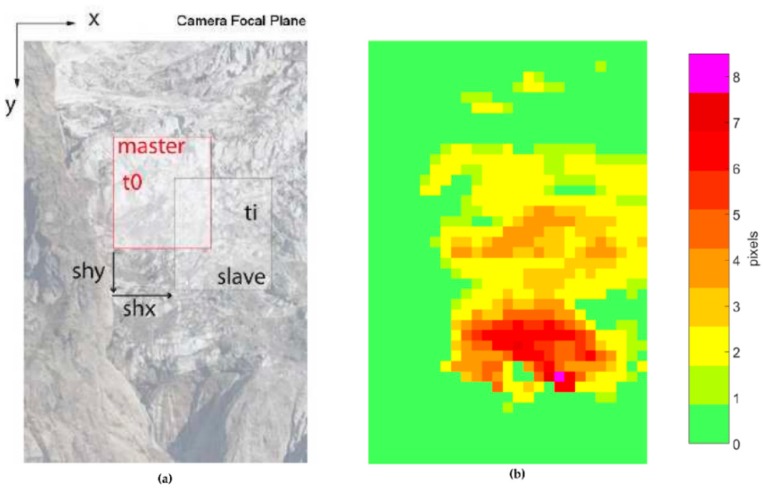
(**a**) Representation of the Pixel Offset iterative algorithm: the tile of the Master image is compared with the same tile of the Slave image. The procedure is applied to all the tiles. (**b**) Example of a result of the Pixel Offset algorithm with the vertical displacement between images computed in pixels.

The algorithm consists of the iteration of the cross-correlation process between different images on pairs of tiles identified by a sliding window (Figure 7a) and it produces displacement maps expressed in px (Figure 7b). Sliding step dimension must be smaller than the tile, for the maximum detectable shift is half the size of the sliding window per the Nyquist criterion. The size of the sliding window is a compromise between the measurement accuracy, for which a small window is preferable, and the measurement precision (i.e., the signal-to-noise ratio). In this case, a large window ensures a better performance [44]. Moreover, the size of the sliding window can significantly affect the measurement [49].

In the current work, the sliding window is a square of 256 px per side, and the moving step is 128 px along both the vertical and horizontal axes. No oversampling factor is adopted because the requested accuracy and the mean error during co-registration are higher than unity and because Casu et al. [49] have proven that this parameter does not affect the results.

#### 4.4.1. Coherence Estimation

Due to many factors (e.g., meteorological events and surface changes), the cross-correlation between images may worsen with time. Therefore, a variable describing the minimum level of correlation necessary to consider tiles comparable is needed. For each pair of tiles, the complex correlation is computed as follows:

(1) $$\gamma =\frac{\langle I_{1}I_{2}^{*}\rangle }{\sqrt{\langle {\left|I_{1}\right|}^{2}\rangle \langle {\left|I_{2}\right|}^{2}\rangle }}$$

where $I_{1,2}$ are the Fourier transforms of the Master and Slave images, respectively; * is the complex conjugate operator, and the angular brackets represent the spatial mean extended over the whole tile. The magnitude of this variable, |γ|, is called *coherence,* as it is related to the signal-to-noise ratio [50] and thus to the quality and reliability of the PO analysis. 

In this study, a threshold of |γ|≥0.75 has been set, not including in the analysis of those pixels with a lower coherence. A further quality check has been adopted, based on a heuristic rule from data observations and physical considerations: tiles showing a negative displacement along the y-axis greater than the system precision (i.e., ±1 px) are rejected because upward movement of the glacier flow is very unlikely.

According to our experience, to achieve a satisfying result, it is important to compare images taken under similar weather conditions and at similar times, i.e., approximately at midday to limit shadow effects.

#### 4.4.2. Displacement Maps

**Figure 8:**
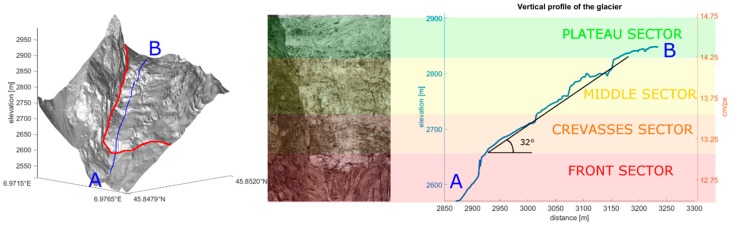
On the left, the DTM produced by the LiDAR survey in June 2014. Blue line identifies the location of the selected vertical profile. The red line highlights the glacier margins. On the right, representation of the vertical profile of the glacier. On average, the glacier lies on a flat plane with a mean slope of 32°. Different colours highlight the sectors of the glacier characterized by different physical features. The right vertical axis reports the pixel dimension increasing upward within the image.

The raw results of the PO application are displacement maps expressed in pixels (Figure 7b). In this section the procedure for the estimation of the metric displacement, i.e., the real displacement amount projected along the surface of deformation, is described. To accomplish this, it is necessary to know the direction of the displacement vectors point-by-point. Thus, it is possible to adopt the DTM surface, and assume that the movement is along the steepest gradient direction. However, in our study, the images are not orthorectified. Thus, in order to explain the method of the metric estimation, we suppose that the glacier movement is parallel to the mean slope of the glacier surface, as observed from the DTM produced by the LiDAR survey in June 2014 (Figure 8). The proposed method is easy to extend to general surfaces.

To estimate the metric displacement, it is necessary to evaluate the Ground Sample Distance (GSD, i.e., the spatial dimension of the pixels projected on ground). This can be achieved using the principles of the optics theory.

Let us consider a surface at a distance  D. It is possible to compute the GSD (cm·px^−1^) as follows [51]:

(2) $$\alpha =\frac{\mathrm{atan}\left(\frac{S}{2f}\right)}{R}$$

(3) $$GSD_{p}=2\cdot D\cdot \tan\left(\alpha \right)$$

(4) $$GSD=\frac{{\mathrm{GSD}}_{p}}{\cos\left(\sigma \right)}$$

where α is the angle of view of one pixel (i.e., the solid angle illuminated by one pixel), S is the sensor size (mm), f is the focal length (mm), R is the camera resolution (px), σ is the angle of incidence on the considered pixel and p stands for perpendicular to the LOS. 

In this case study, D and σ are unknown, as the images are not georeferenced. Thus, the method is applied approximating the movement direction to a flat plane, and D and σ are estimated with simple geometric calculus. The portion of glacier illuminated by the camera lies on a plane with the horizontal axis orthogonal to the LOS and a slanted vertical axis. A section profile derived by an aerial laser-scanner acquisition yielded a mean glacier slope of 32° (with respect to the horizontal), and the camera elevation angle is approximately 6°.

The horizontal GSD varies with the distance, in accordance with Equation (3). However, because the LOS in not orthogonal to the glacier, the vertical GSD is corrected with Equation (4). This system scheme involves a GSD that linearly increases upward in both the vertical and horizontal dimensions. 

In Table 3, we report the geometrical and optical variables of the system.

The obtained pixel size is the actual GSD of the image projected along the surface of the glacier. The main error during the procedure is the approximation of the deformation surface parallel to the mean slope of the glacier. This can cause an overestimation of the actual GSD in steep areas (e.g., ice walls) and underestimation in gently sloping areas (e.g., plateau). It was not possible to estimate the actual pixel-by-pixel GSD [31,44] because it would require an ultra-high-resolution DTM (comparable to the GSD) up-to-date at the acquisition time of each image (necessitating at least one DTM per season) as the glacier evolution causes great surface changes within short periods of time. Moreover, the deformation surface could not coincide with the glacier surface, as usually the glaciers are affected by the creeping process, which involves a movement direction steeper than the glacier surface [52,53,54]. By way of example, if the glacier movement direction is 90°, the GSD is approximately 5 cm; if the movement direction is half of the mean slope, the GSD is approximately 35 cm. 

#### 4.4.3. EAPOE

To support the management of a large dataset for the post-processing analysis, a graphical user interface tool for Matlab, called Effective Analysis for Pixel Offset Evaluation (EAPOE), has been developed. EAPOE creates a graphical representation of the maps of cumulative vertical displacement, mean vertical velocity and the fraction of data with acceptable coherence for the chosen period. In addition, the user can select a tile of the map to show the time series of cumulative vertical displacement and daily vertical velocity. 

Moreover, by selecting one point of the temporal line, EAPOE displays the photographs of the current, the previous and the next day zoomed in on the neighbourhood of the selected tile.

This tool allows for fast and effective post-processing analysis, combining numerical and visual information. The graphical interface is designed to be user-friendly, which makes it possible to introduce EAPOE on open platforms, such as web sites.

**Figure 9:**
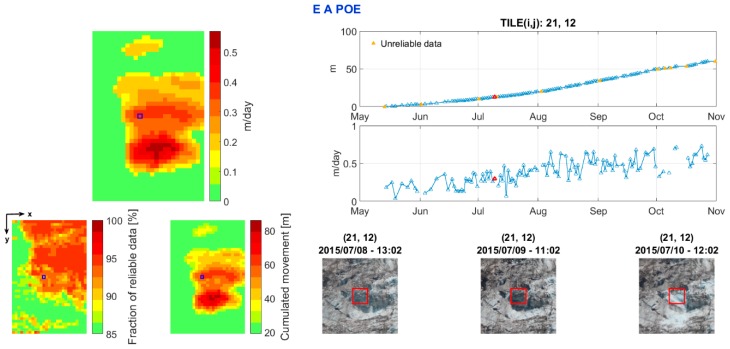
EAPOE output. On the left, there is a mean vertical velocity map (**top**), a fraction of days with acceptable coherence map (**bottom left**) and a cumulative vertical displacement map (**bottom right**). The time series of cumulative displacement and daily velocities of the selected tile are plotted on the right. At the bottom, the images show the glacier evolution.

In Figure 9, example of the output of EAPOE is reported. In the time series, the data under the coherence threshold (or outside the acceptable movement interval) are shown in yellow. As an example, a tile belonging to the middle sector is chosen (highlighted in blue in the maps on the left side). The selected tile shows a loss of coherence between the 9 and 10 of July 2015 due to an icefall, which can be observed in the photographs at the bottom.

## 5. Results

In the following, only the vertical displacement results are presented, as the horizontal deformations are negligible in the considered portion of glacier. As previously mentioned, the analysis is made using one image per day from approximately midday to limit the effects of shadow.

### 5.1. Displacement Time Series and Maps

**Figure 10:**
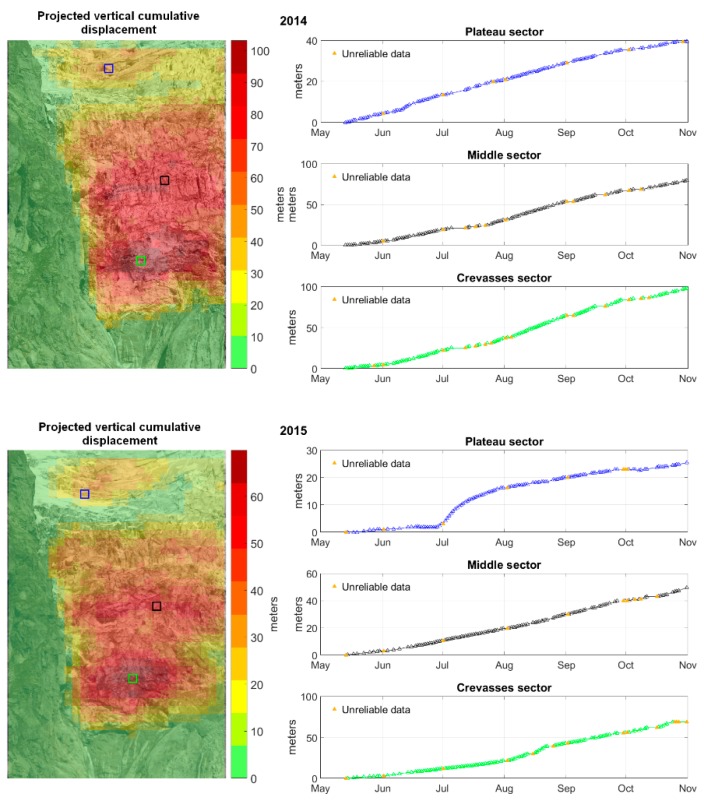
Projected vertical cumulative displacement maps for 2014 and 2015 and time series of displacement of different sectors. From the comparison one can note the behaviour changes and anomalies during time and space and identifies critical data.

The cumulative deformation maps are compared between the years 2014 and 2015, within the period 13 May to 31 October. Pixels representative of the different sectors are selected to display the time series of displacement, shown in Figure 10. As a result, it is possible to observe a great difference between the years in terms of total displacement. In contrast, the patterns of deformation appear quite similar. The pixel time series present different behaviour between years and between sectors. 

This comparison shows the capabilities of the PO method in terms of the analysis of the glacier kinematics. The results document possible inter-annual changes and the yearly glacier behaviours of different regions, thereby potentially leading to the identification of break-off precursors or anomalies.

### 5.2. Sectors

The post-processing analysis with EAPOE confirms the visual definition of the four different sectors of the glacier based on their different behaviours. Figure 8 shows a representation of the study area and a representation of the vertical section with an estimate of the resolution in cm·px^−1^ of different sectors. The relatively slow velocity recorded in the *plateau sector* is partially due to the gentle slope, which induces an underestimation of the ground resolution (due to the optical incidence). Therefore, the PO analysis computes only small displacements. The *front sector*, characterized by recurring icefalls, is the most difficult to analyse with the PO analysis due to the loss of coherence related to the collapses.

### 5.3. Comparison between PO and GB-SAR Measurements

In this section, we present a comparison of the glacier deformations estimated with the PO method and with GB-SAR. The dimensions of the pixels captured by the two devices are quite different. With the PO method, the dimensions of a single tile of the deformation maps are approximately 14 × 5.7 m^2^ (vertical and horizontal, respectively), whereas the pixel dimensions of the GB-SAR are approximately 0.5 × 10 m^2^. Moreover, because the results are not geo-referenced in a common reference system, the comparison is rather qualitative. Nevertheless, the movement pattern is similar for both techniques and, considering the great difference in resolution, it is possible to compare the mean displacement of entire sectors, which are recognizable within the deformation maps of both devices.

We note that the GB-SAR analysis is preliminary and that the GB-SAR acquires better data in very steep areas, where the angle of the movement direction is probably greater than the mean slope. For these reasons, the GSD is estimated for an angle of 40°, which is the mean slope in the vicinity of the ice walls.

The comparison between the PO results and GB-SAR measurements during the period from 4 September 2015 and 27 September 2015 is performed and shown in Table 4. For this comparison, the mean daily velocities and the standard deviations computed for each sector are reported for both methods, except for the plateau sector, which was not viewed by the GB-SAR. 

The agreement is good, i.e., within the error bars, although the values of the PO are slightly larger. This is likely due to an underestimation of the slope of the deformation surface. Future work will focus on the coupling of the data from both devices.

## 6. Discussion

In this paper, we have presented a low-cost monitoring and processing chain with an optical system for the evaluation of glacier deformations. The described procedure includes all the steps necessary to achieve this aim, including the installation of the monitoring station, the data acquisition, and the visual and computer analysis with the Pixel Offset technique. This work aims to assess the performances of the PO analysis and to define a processing chain for the collection and analysis of photographic data. 

The combination of remote sensing and image processing approaches based on low-cost equipment allows us to study dangerous areas where in situ instrumentations cannot be installed. Furthermore, optical data permits a visual approach for the interpretation and control of the results. Our experience shows that the visual analysis allows the study of the flow of the glacier both in term of displacement and dynamics (e.g., water discharge, crevasse opening and closing, and collapses). Some of these phenomena are interesting but difficult to describe quantitatively. Therefore, a visual analysis of the images can provide information that is not detectable with the PO technique. In contrast, the PO method produces quantitative results, which are necessary for a deep understanding of the glacier behaviour. Moreover, PO results show good agreement with data recorded by a GB-SAR system.

Applying the PO algorithm, some sources of uncertainty can affect the measurements. Among these, the most important are as follows. (i) Camera vibrations and thermal effects during the co-registration phase (these errors have been statistically analysed and quantified); (ii) shadow effects and extreme light conditions and (iii) icefalls and the presence/absence of snow (these uncertainties are addressed in the coherence evaluation, although a heuristic empirical threshold may be required to exclude the data that satisfy the coherence threshold but are still subject to great errors); (iv) changes in the slope, which affect the pixel-to-metric conversion. In the current work, the deformation surface is approximated to be parallel to the mean glacier slope, but for the comparison with GB-SAR data, a local slope is used for the GSD estimation.

The current work focuses on a limited portion of the glacier because it aims to define a procedure for the PO analysis rather than to study the glacier kinematics. Many factors were involved in the choice of the studied area, such as the possibility of placing the monitoring station in front of it. Moreover, the LOS is perpendicular to the maximum gradient direction; thus, the movement of the glacier in the images is maximized in the vertical dimension and negligible in the horizontal dimension. The dimension of the area has been set to maximise the pixel resolution.

In this paper, we have proposed a method for the metric conversion of the observed pixel displacement. The main issue is the knowledge of the actual direction of the deformation vectors, which do not coincide with the mean slope because of local changes in the gradient and perhaps not even with the glacier surface because of creeping processes [52,53,54]. The proposed method can be easily applied to orthorectified images in general.

Future work will focus on two main lines of inquiry. The first will focus on processing improvements, including process automation, e.g., the identification of icefalls. This could lead to the realization of a completely automatic remote sensing system, able to relatively inexpensively monitor glacier deformation even in dangerous and difficult-to-reach areas. The second research line will focus on a practical application of the current PO system to the entire glacier (of which there are already three years of data) as a means to analyse the geophysics of the glacier. The use of this systems might be useful for improving the understanding of glacier kinematics and for the evaluation of numerical models. According to the state-of-the-art knowledge [3,55], there is no way to predict break-off events with significant forewarning by means of measurable parameters. Nevertheless, according to modelling applied to the analysis of historical cases, some morphological evidence can be considered predictive of potential instabilities, such as (i) a sudden increase or decrease in water runoff, (ii) rapid advance of the glacier tongue, or (iii) increase fracturing of the glacier tongue [43]. According to our experience, these elements might be detected using the presented system, which is a monitoring system that is able to analyse daily evolution but currently cannot be considered ready for civil protection purposes and/or early warning applications. 

## 7. Conclusions

In the last decade, advances in hardware and software have led to the design of optical and visual technologies for the quantitative monitoring of gravitational processes [24]. Different approaches have been proposed, most commonly involving satellite [18,19,25] and airborne images [14,15]. More recently, ground-based photogrammetry began to be applied for Earth science purposes [29,44]. This approach has two main advantages: (i) low-cost equipment and (ii) the possibility of implementing continuous and long-lasting survey campaigns.

In this paper, we have presented a low-cost monitoring and processing chain based on an optical system for the evaluation of glacier deformation. Data have been acquired with an hourly frequency since September 2013 by the monitoring station, which has proven to be rugged enough to withstand the mountain weather. Such a large amount of data allows for a visual morphological analysis of the glacier. This analysis is able to recognize the most important features that characterized the observed phenomena. For the evaluation of the glacier displacement, a Pixel Offset algorithm has been developed to process one image per day. For an easier and faster post-analysis, a Matlab GUI has been developed to produce a user-friendly application with which non-expert end-users can process entire datasets.

We proposed a method for the pixel-to-metric conversion, and the observed pixel shifts between different images have been projected along the maximum gradient direction of the mean slope of the glacier surface. The acquired dataset allows for the first time the possibility of measuring the glacier evolution, in particular during the warm season, when the displacement rate is higher.

The results have been compared with GB-SAR surveys, adopting local mean slope for the metric conversion. The results of the comparison are in agreement, considering the errors of the PO algorithm. This demonstrates the reliability of the developed method in terms of measuring glacier deformations.

Future improvements might consist of image georeferencing, a better description of the glacier surface for 3D projection, and the automatic detection of areas subject to collapses. 

## Figures and Tables

**Table 1:** Main characteristics of the cameras mounted in the monitoring station.

Monitoring Module	Camera	Sensor/Resolution	Aperture Lens	Camera Lens	Focus	ISO
ZOOM	Canon EOS 600D	CMOS APS-C/5184 × 3456 px	f/8	(120–400 mm) Settled at 297 mm	Manual ∞	200
WIDE	Canon EOS 100D	CMOS APS-C/3456 × 5184 px	f/8	(100–300 mm) Settled at 120 mm	Manual ∞	200
Webcam	Canon Powershot	CMOS 8 Mpx	Auto f/2.6–5.5	(28–110 mm)	Auto 9 points	100

**Table 2:** Summary of the acquisitions during different visibility conditions. During winter, the system is frequently subjected to power failures and/or extreme weather conditions, during which data acquisition is impossible. In summer, the system runs almost continuously.

	Conditions	Number of Days	Fraction
Acquired images	Good visibility	414	40%
	Poor visibility	235	23%
Images not acquired	Technical problems	69	7%
	Power failure due to snow coverage	305	30%

**Table 3:** Environmental and optical parameters of the system.

Mean distance (m)	3800	LOS angle of incidence	64°
Horizontal AOV	2.87°	Vertical AOV	4.3°
Vertical GSD at the bottom (cm)	12.49	Vertical GSD at the top (cm)	14.79
Horizontal GSD at the bottom (cm)	5.35	Horizontal GSD at the top (cm)	5.68

**Table 4:** Comparison of mean daily velocities between the Pixel Offset method and GB-SAR. The acquisition time refers to the period from 4 September 2015 to 27 September 2015. The error is given as the standard deviation.

Sectors	GB-SAR (cm/day)	Pixel Offset (cm/day)
*Front sector*	29.2±3.8	31.4±10.3
*Crevasses sector*	23.0±2.6	29.9±4.5
*Middle sector*	20.8±3.9	26.0±7.2
